# A discovery plasma proteome signature in military personnel with chronic PTSD symptoms

**DOI:** 10.3389/fpsyt.2026.1898600

**Published:** 2026-09-15

**Authors:** Arum Lim, Claire Robey, Kimbra Kenney, Sijung Yun, Joseph Yun, John Alice, Heather Dark, Jessica M. Gill, Sara M. Lippa

**Affiliations:** 1 School of Nursing, Johns Hopkins University, Baltimore, MD, United States; 2 National Intrepid Center of Excellence, Bethesda, MD, United States; 3 Uniformed Service University of Health Sciences (USUHS), Bethesda, MD, United States; 4 Henry Jackson Foundation, Bethesda, MD, United States; 5 School of Medicine, Johns Hopkins University, Baltimore, MD, United States

**Keywords:** biomarker, discovery, military personnel, pathway analysis, post-traumatic stress disorder, proteomics

## Abstract

**Introduction:**

Post-traumatic stress disorder (PTSD) is highly prevalent among U.S. service members and veterans (SMV) and has lasting impacts on health and well-being. However, the biological underpinnings of PTSD remain poorly characterized. This study aimed to discover novel candidate proteins and protein pathways associated with PTSD using unbiased, high-throughput proteomics profiling.

**Methods:**

A cross-sectional study was conducted using a subset of SMV participants from a clinical cohort undergoing evaluations at the National Intrepid Center of Excellence, Walter Reed National Military Medical Center, who consented to a research blood draw and use of their clinical data in research. The cohort with available blood samples was classified into PTSD-Present and PTSD-Absent groups based on the PTSD Checklist-Civilian Version. Olink high-throughput proteomic profiling examined 5400 human plasma proteins, and differentially expressed proteins were examined. Ingenuity pathway analysis was used to identify proteomic pathways among the significant differentially expressed proteins between the PTSD-Present and PTSD-Absent groups.

**Results:**

We included 208 samples in our analysis, 126 with and 82 without PTSD. We identified 366 proteins that were significantly differentially expressed between groups, with the 3 most significant being LMOD2 (Leiomodin-2), ATP5F1D (ATP synthase δ-subunit), and CASKIN1 (CASK-interacting protein). Extracellular matrix organization pathways and Vascular Endothelial Growth Factor (VEGF) signaling were downregulated in PTSD, suggesting possible vascular inflammation and remodeling. Data-driven protein networks suggest reduced immune cell activation.

**Discussion:**

Determining differential protein expression and identifying the associated protein pathways linked to PTSD may provide new insights into the biological basis of chronic PTSD symptoms and help identify novel candidate protein biomarkers of PTSD and PTSD symptoms for validation in separate and larger cohorts. From this discovery cohort, we report that elevated PTSD symptoms may be associated with downregulation of extracellular matrix organization, inflammation signaling, and vascular remodeling pathways. Future research is necessary to validate this novel group of biomarkers and pathways.

## 1 Introduction

Post-traumatic stress disorder (PTSD) is highly prevalent among U.S. Veterans, with a lifetime PTSD prevalence of 9.4% (1), compared to 6.1% in civilians (2). PTSD is associated with increased morbidity and mortality, including increased risk of the development of serious and often chronic health conditions, such as cardiovascular and pulmonary disease (3), as well as suicidality/completed suicide and substance use disorders (4). It also frequently negatively impacts daily functioning (5). The increased morbidity and mortality risk may be related to alteration of biological pathways previously identified in individuals with PTSD, such as dysregulated metabolism (6, 7), increased inflammation (8), and brain volume reduction (9). Although chronic PTSD symptoms may underpin these biological alterations, few studies have examined the pathophysiological processes associated with chronic PTSD symptoms using high-sensitivity proteomics. This approach enables broad, unbiased measurement of human protein expression and allows differentially expressed proteins to be mapped to established, canonical signaling pathways, offering insight into biological pathways associated with chronic symptom burden.

Several studies have examined the association between PTSD and inflammatory markers (8, 10, 11). Specifically, higher concentrations of IL-6 and IL-10 are positively associated with PTSD symptoms in community-based samples, as well as in service members and veterans (SMVs) (10–12). Further, a comprehensive systematic review demonstrated that IL-1β, IL-2, IL-6, interferon-γ, tumor necrosis factor-α, C-reactive protein, and white blood cell count were higher in participants with PTSD compared to controls (8). Numerous biomarker studies have been published with individuals with PTSD, particularly among SMVs; however, the majority have examined a limited number of candidate biomarkers (most commonly < 10) selected based on associations with comorbid conditions, such as traumatic brain injury (TBI) and dementia (13), rather than PTSD symptom burden itself. This candidate-driven approach limits discovery potential and may introduce bias by prioritizing biologically adjacent conditions over PTSD-specific pathophysiology. To date, validated findings are limited and variable among studies that have examined associations between blood biomarkers and PTSD (14, 15).

Given the lack of consistent findings among traditionally measured blood biomarkers in samples of individuals with PTSD (14, 15), we hypothesized that leveraging large-scale (>5,000), unbiased proteomics and examining established canonical, human biological pathways may not only introduce novel potential candidate markers that are associated with the biological mechanisms of PTSD, but may also elucidate proteomic pathways that underlie persistent PTSD symptoms. Previous studies that used unbiased proteomics in other behavioral health disorders reported unique proteins and pathways associated with depression, including upregulation of Macrophage Inflammatory Protein-1ß (CCL4), C-X-C motif chemokine ligand 5 (CXCL5), C-X-C motif chemokine ligand 11 (CXCL11), and Interleukin-18 (IL-18) in depression (16), and a link between Butyrophilin Subfamily 3 Member A2 (BTN3A2) and depression onset (17). However, there is limited data in proteomic and pathway-based approaches in PTSD. To address this gap, we undertook a cross-sectional analysis of Olink in SMVs with and without PTSD. We hypothesized that proteomic analysis among a homogenous cohort of SMVs with and without high PTSD symptoms may similarly identify novel candidate proteins and pathways associated with PTSD that haven’t yet been identified in earlier studies of limited candidate proteins. Further, we hypothesized that pathway analysis of the most significantly over- or under-expressed proteins may identify or help us to better understand the biological underpinnings of chronic PTSD symptomology.

## 2 Materials and methods

### 2.1 Study design and sample

The present study employed a cross-sectional design using a subset of participants from the parent cohort study focused on neuroimaging data collection and analysis, which included a blood draw and self-report measures. Of the 461 participants who provided a blood sample in the parent study, 33 were excluded as non-deployed controls and 8 for not completing the PCL-C (n=420). An additional 187 participants were excluded for not meeting classification criteria for either the PTSD-Present or PTSD-Absent group (n=233), and 7 were excluded to achieve age-matching between groups (n=226). Olink proteomic data were unavailable for the remaining 18 participants, yielding a final analytic sample of 208. Most (n=171, 82.2%) participants were active duty U.S. service members undergoing clinical evaluation and treatment in the intensive outpatient program (IOP) six or more months following mild TBI (mTBI) or associated health conditions at the National Intrepid Center of Excellence (NICoE) at Walter Reed National Military Medical Center (WRNMMC) in Bethesda, MD. Seventeen participants (8.2%) were outpatients at NICoE with history of mTBI, and 20 participants (9.6%) were recruited as deployed controls without any TBI history. Participants were prospectively enrolled in a WRNMMC IRB-approved Neuroimaging study (Protocol # 20337) of WRNMMC military healthcare beneficiaries that included an optional research blood draw and the use of the participant’s imaging and clinical data for research purposes. All participants included in this analysis provided informed consent. A history of major neurologic or psychiatric conditions, including psychosis, stroke, multiple sclerosis, or spinal cord injury, was exclusionary for enrollment in the overall study. This research was approved by the Institutional Review Board of WRNMMC, is compliant with the Health Insurance Portability and Accountability Act (HIPAA) and was conducted in accordance with the Declaration of Helsinki guidelines.

### 2.2 Measurements

PTSD symptoms were assessed with the PTSD Checklist-Civilian (PCL-C) (18) to capture the broadest range of potential traumatic stressors and events not restricted to combat/deployment-related events. The PCL-C has demonstrated reliability and validity in both civilian and veteran populations (19), and comparability with PCL-5 (20). Participants were included in the PTSD-Present group if their report met the Diagnostic and Statistical Manual of Mental Disorders-4th edition (DSM-4) criteria based on moderate or higher symptom endorsement (i.e., at least one moderate or higher re-experiencing symptom, two moderate or higher avoidance symptoms, and two moderate or higher hyperarousal symptoms) and they also had a total score of 45 or higher on the PCL-C. Participants were included in the PTSD-Absent group if they did not meet DSM-4 criteria based on moderate or higher symptom endorsement and their PCL-C total score <30. The two groups (n=126, PTSD-Present; n=82, PTSD-Absent) were matched on sex, age, race, and military rank (officer or enlisted) to minimize potential confounding factors that could affect protein expression.

### 2.3 Blood sample preparation and proteomics profiling

Non-fasting blood samples were collected with plastic plasma-separating tubes, processed within an hour, and stored at -80 °C. Proteomic profiling was performed using the Olink Explore High Throughput (HT) Proximity Extension Assay (PEA)-based technology, enabling multiplex quantification of approximately 5,400 protein biomarkers. The assay relies on pairs of antibody binding and Deoxyribonucleic acid (DNA)-based detection, providing high specificity and sensitivity in complex biological matrices, such as plasma or serum. For this study, plasma samples were aliquoted into pre-assigned wells and transferred to sample source plates, followed by automated dilution. During incubation, PEA™ probe pairs bind to their target proteins, allowing the attached DNA oligonucleotides to come into proximity for downstream ligation and amplification. Following incubation, hybridized oligonucleotides were then extended to generate unique DNA barcodes, which were subsequently amplified by a Polymerase Chain Reaction (PCR). Sample-specific indexes were added to enable pooling of amplicons, producing libraries containing assay-specific barcodes, sample indexes, and Illumina sequencing adapters. After library purification and quality check, samples were then pooled into the final library and sent for next-generation sequencing on the Illumina platform. The relative concentration of each biomarker, based on matched counts, was calculated using the Olink Explore HT software. Protein expression was normalized using intensity-based normalization to generate Normalized Protein eXpression (NPX) values to measure relative changes between samples.

### 2.4 Differential protein expression analysis

We used the Wilcoxon rank-sum test from the *OlinkAnalyze* package (version 4.0.1) in R (version 4.4.3) to compare NPX levels of 5400 proteins between the two groups. To account for multiple testing, p-values from the Wilcoxon rank-sum tests were adjusted using Benjamini-Hochberg’s False Discovery Rate (FDR) method. As the final sample size was determined based on data availability, we conducted a *post-hoc* power analysis using the *Olink Study Size Calculator*, based on the empirical pooled standard deviation across the panel (SD = 0.86) and the smaller of the two group sizes (n=82, PTSD-Absent), for a conservative estimate. Using a Bonferroni-corrected significance threshold (α=0.05 across the panel), the study had 70-96% power to detect effect sizes of Cohen’s f=0.4 to 0.5.

### 2.5 Ingenuity pathway analysis

Ingenuity pathway analysis (IPA) was used to examine previously established canonical pathways, predefined groups of genes involved in a biological process. Proteins differentially expressed between the two groups with unadjusted p-values <0.05 were uploaded to IPA for Core Analysis (QIAGEN Inc., https://digitalinsights.qiagen.com/IPA) for discovery purposes. Canonical pathway analysis shows the most significant signaling pathways that include differentially expressed proteins, with z-scores reflecting predicted activation (>0) or inhibition (<0) based on the directionality of observed protein expressions that belong in the pathways, with FDR-adjusted p-values from Fisher’s exact test to determine if a pathway is significantly enriched by the differentially expressed proteins. In addition to demonstrating biological connections among differentially expressed proteins, protein networks were generated with nodes and lines based on previously established pathways from published manuscripts, textbooks, or canonical data in the knowledge base of human genes. The color of the nodes indicates the level of differential expression (red for upregulation, green for downregulation) and the predicted activation status (orange for activated, blue for inhibited, gray for not predicted), with higher color intensity indicating higher levels of expression or prediction.

## 3 Results

The final sample consisted of 208 participants, including 126 individuals with significant PTSD symptoms (PTSD-Present) and 82 with minimal or no PTSD symptoms (PTSD-Absent). There were no group differences in age (p=.163), years of education (p=.711), sex (p=1.0), officer status (p=.134), or military branch (p=.234). All participants were self-reported as White/Caucasian origin. As expected, based on group selection criteria, PCL-C total score was significantly higher in the case group (Mean=57.7, SD = 8.7) compared to the control group (Mean=23.3, SD = 4.3; p<.001). Mild TBI history was significantly higher in the case group than the control group (79.4% vs 54.9%, p<.001). Demographic and military characteristics are summarized in **Table 1**.

**Table 1:** Demographics and military characteristics.

Characteristic	PTSD-Absent		PTSD-Present		Welch F	p
	(n=82)		(n=126)			
	Mean	SD	Mean	SD		
Age	39.56	6.69	40.79	5.33	1.97	0.163
Years of Education	15.43	2.28	15.31	2.12	0.14	0.711
PCL-C Total	23.31	4.25	57.71	8.72	1396.10	<.001
	n	%	n	%	Chi-Square	p
Male	79	96.3	120	95.2		1.0*
Officer^	26	31.7	29	23.0	2.25	0.134
Branch^		0.0		0.0	4.27	0.234
Air Force	12	14.6	12	9.5		
Army	26	31.7	32	25.4		
Marines	3	3.7	11	8.7		
Navy	40	48.8	71	56.3		
mTBI history	45	54.9	100	79.4	15.12	<.001

*Fisher’s Exact Test.

^Within the control group, information was missing for 2 individuals regarding rank and one individual regarding Branch.

Differential protein expression analysis identified 366 proteins that were differentially expressed between the two groups based on the unadjusted p-value cutoff (p<0.05). After FDR correction, none of the individual proteins remained significant. The top 20 proteins with an absolute value log2 fold change (|log2FC|)>0.5 and ordered by p-value are presented in **Table 2**, including LMOD2 (Leiomodin-2), ATP5F1D (ATP synthase δ-subunit), and CASKIN1 (CASK-interacting protein).

**Table 2:** Top 20 differentially expressed proteins with p-value<0.05, |Log2FoldChange|>0.5.

Protein	Primary function	log2FC	P-value
LMOD2 (Leiomodin-2)	Actin nucleation/sarcomere contractility	-0.533	4.64E-04
ATP5F1D (ATP synthase δ-subunit)	Proton gradient coupling/adenosine triphosphate (ATP) synthesis	-0.793	9.34E-04
CASKIN1 (CASK-interacting protein)	Postsynaptic scaffolding/synaptic transmission	-1.166	1.38E-03
NLGN1 (Neuroligin-1)	Neurexin binding/excitatory synapse maturation	-0.565	1.40E-03
ADI1 (Acireductone dioxygenase 1)	Acireductone cleavage/methionine recycling	-0.553	2.27E-03
RTF2 (Replication termination factor 2)	Replication fork stabilization/genome integrity	0.688	2.34E-03
PASD1 (PAS domain-containing protein 1)	Transcriptional co-regulation/circadian gene expression	-0.725	4.31E-03
VPS41 (Vacuolar protein sorting 41)	Endosome tethering/lysosomal cargo delivery	0.749	6.16E-03
VAX2 (Ventral anterior homeobox 2)	Retinal transcription factor/photoreceptor axon guidance	0.982	9.78E-03
TRAF3IP1 (TRAF3-interacting protein 1)	Intraflagellar transport/ciliogenesis and NF-κB modulation	-0.684	1.01E-02
ZFX (Zinc finger X-chromosomal protein)	Transcriptional activation/stem cell proliferation	0.700	1.12E-02
MN1 (Meningioma 1)	Retinoic Acid Receptor (RAR) and Retinoid X Receptor (RXR) co-activation/retinoic acid-driven development	0.895	1.24E-02
ARPP21 (cAMP-regulated phosphoprotein 21)	Calmodulin inhibition/dopamine-Protein Kinase A (PKA) signal amplification	0.666	1.30E-02
PCARE (Photoreceptor cilium actin regulator)	Ciliary F-actin assembly/outer segment disk biogenesis	-0.511	1.41E-02
TNS4 (Tensin-4)	Integrin-actin bridging/cell migration	0.500	1.42E-02
CCDC7 (Coiled-coil domain-containing protein 7)	Cytoskeletal scaffolding/cell division (function not fully characterized)	0.626	1.53E-02
KIAA2026	Unknown mechanism and effect	-0.838	1.70E-02
CDK5RAP3 (CDK5 regulatory subunit-associated protein 3)	Cyclin-dependent kinase 5 (CDK5)/NF-κB modulation/cell cycle and inflammatory control	-0.602	1.81E-02
TIA1 (T-cell intracellular antigen 1)	Stress granule formation/translational silencing under stress	-0.706	2.07E-02
CFAP20 (Cilia and flagella associated protein 20)	Dynein arm assembly/axonemal motility	0.521	2.17E-02

Proteins are ordered by p-values.

For exploratory analyses, proteins with an unadjusted p-value <0.05 were used for canonical pathway analysis. Of the 366 significant proteins, 357 were mapped to the IPA reference dataset, including 231 down-regulated and 126 up-regulated proteins. Canonical pathways with FDR-adjusted p<0.05 and |Z-score|≥2 were counted as significant and presented in **Table 3**. Extracellular matrix (ECM) organization and wound healing signaling pathways were considered to be inhibited, given the observed directionality of changes in protein expression. Following this, there was an indication of possible downregulation of the cyclic guanosine monophosphate (GMP)-adenosine monophosphate (AMP) synthase–stimulator of interferon genes (CGAS-STING) signaling pathway, a crucial innate immune mechanism. Downregulation of Vascular Endothelial Growth Factor (VEGF) signaling and VEGF family ligand-receptor interaction was also observed, suggesting possible altered vascular remodeling.

**Table 3:** Significant canonical pathways with FDR<0.05, |Z score|>=2.

Canonical pathway	Adjusted p-value	Activation z-score	Overlap	Molecules
Extracellular matrix organization	4.73E-05	-2.31	11.21%	ASPN, BCAN, COL2A1, COL4A1, COL9A1, ITGAV, ITGAX, NRXN1, PTPRS, SERPINE1, SPARC, TNN
Wound Healing Signaling Pathway	1.13E-03	-3.87	6.28%	COL2A1, COL4A1, COL9A1, IL11, IL17D, IL17F, IL1RL2, IL3, IL33, NFKB1, PDGFD, PF4, RRAS, TNFSF12, TNFSF9
CGAS-STING Signaling Pathway	1.19E-03	-3.32	8.27%	CDKN1A, EIF2AK3, IL11, IL17D, IL17F, IL3, IL33, NFKB1, TFAM, TNFSF12, TNFSF9
Hepatic Cholestasis	0.01	-2.89	5.56%	IL11, IL17D, IL17F, IL1RL2, IL3, IL33, LY96, MAP3K4, NFKB1, PRKCQ, TNFSF12, TNFSF9
Glycation Signaling Pathway	0.01	-2.89	5.38%	COL4A1, EIF2AK3, IL11, IL17D, IL17F, IL3, IL33, NFKB1, PRKCQ, RRAS, TNFSF12, TNFSF9
HMGB1 Signaling	0.01	-2.45	6.29%	IL11, IL17D, IL17F, IL3, IL33, NFKB1, RRAS, SERPINE1, TNFSF12, TNFSF9
Signaling by VEGF	0.02	-2.83	7.48%	CTNNA1, FLT1, ITGAV, MAPKAPK2, NCK2, NOS3, NRP1, NRP2
VEGF Family Ligand-Receptor Interactions	0.02	-2.24	8.33%	FLT1, NOS3, NRP1, NRP2, PLA2G10, PRKCQ, RRAS
FXR/RXR Activation	0.02	3.00	5.65%	EIF2AK3, GSTP1, IL11, IL17D, IL17F, IL3, IL33, NFKB1, TNFSF12, TNFSF9
Pulmonary Fibrosis Idiopathic Signaling Pathway	0.02	-2.50	4.35%	ADAMTS1, COL2A1, COL4A1, COL9A1, IL11, ITGAV, NFKB1, NOTCH1, PDGFD, PLAU, PTEN, RRAS, SERPINE1, WNT9A
Cardiac Hypertrophy Signaling (Enhanced)	0.02	-2.50	3.58%	HDAC5, IL11, IL17D, IL17F, IL1RL2, IL3, IL33, ITGAM, ITGAV, ITGAX, MAP3K4, MAPKAPK2, NFKB1, PRKCQ, PTEN, RRAS, TNFSF12, TNFSF9, WNT9A
Cachexia Signaling Pathway	0.03	-3.21	3.89%	EIF2AK3, IL11, IL17D, IL17F, IL1RL2, IL3, IL33, INHBB, LIFR, NFKB1, PRKCQ, PTEN, TNFSF12, TNFSF9
Hematoma Resolution Signaling Pathway	0.04	2.11	4.37%	IL11, IL17D, IL17F, IL3, IL33, ITGAM, LRP1, NFKB1, SOD3, TNFSF12, TNFSF9
NOD1/2 Signaling Pathway	0.04	-2.33	5.03%	IL11, IL17D, IL17F, IL3, IL33, MAPKAPK2, NFKB1, TNFSF12, TNFSF9
NAFLD Signaling Pathway	0.04	-3.16	4.63%	EIF2AK3, IL11, IL17D, IL17F, IL3, IL33, NFKB1, PRKCQ, TNFSF12, TNFSF9
Macrophage Classical Activation Signaling Pathway	0.04	-3.00	5%	IL11, IL17D, IL17F, IL3, IL33, LY96, NFKB1, TNFSF12, TNFSF9
Integrin cell surface interactions	0.04	-2.45	7.06%	COL2A1, COL4A1, COL9A1, ITGAM, ITGAV, ITGAX
Signaling by PDGF	0.04	-2.24	8.62%	COL2A1, COL4A1, COL9A1, NCK2, PDGFD
Interleukin (IL)-17 Signaling	0.04	-3.00	4.89%	IL11, IL17D, IL17F, IL3, IL33, NFKB1, RRAS, TNFSF12, TNFSF9
Pathogen Induced Cytokine Storm Signaling Pathway	0.04	-3.61	3.82%	COL2A1, COL4A1, COL9A1, IL11, IL17D, IL17F, IL1RL2, IL3, IL33, NFKB1, PF4, TNFSF12, TNFSF9
PAK Signaling	0.04	-2.00	5.98%	DSCAM, ITGAM, ITGAV, ITGAX, NCK2, PDGFD, RRAS
Autism Signaling Pathway	0.04	-2.11	3.99%	IL11, IL17D, IL17F, IL3, IL33, NLGN1, NRXN1, PTEN, RRAS, TNFSF12, TNFSF9, WNT9A
Neutrophil degranulation	0.04	-3.50	3.35%	ACP3, ADGRE3, CD93, CEP290, CNN2, GSN, GSTP1, ITGAM, ITGAV, ITGAX, NFKB1, PLAU, PTPN6, RAB37, TIMP2, VAT1
NCAM signaling for neurite out-growth	0.04	-2.24	7.94%	CNTN2, COL2A1, COL4A1, COL9A1, GDNF

Pathways are ordered by FDR (false discovery rate) adjusted p-values.

CGAS-STING, cyclic guanosine monophosphate (GMP)-adenosine monophosphate (AMP) synthase–stimulator of interferon genes, HMGB1, High-mobility group box 1; VEGF, Vascular Endothelial Growth Factor; FXR/RXR, Farnesoid X receptor (FXR) and Retinoid X receptor (RXR); NOD1, Nucleotide-binding oligomerization domain-containing protein 1; NAFLD, Nonalcoholic Fatty Liver Disease; PDGF, Platelet-Derived Growth Factor; PAK, p21-activated kinase; NCAM, Neural Cell Adhesion Molecule.

**Figure 1** illustrates a data-driven protein network. Observed down-regulated proteins, including Sodium/potassium-transporting ATPase subunit beta-1 (ATP1B1), Tripartite motif-containing 69 (TRIM69), and Lymphocyte Antigen 96 (LY96), were collectively associated with suppression of NFκB signaling and predicted inhibition of interferon alpha, suggesting reduced immune cell activation and dampened inflammation response.

**Figure 1:**
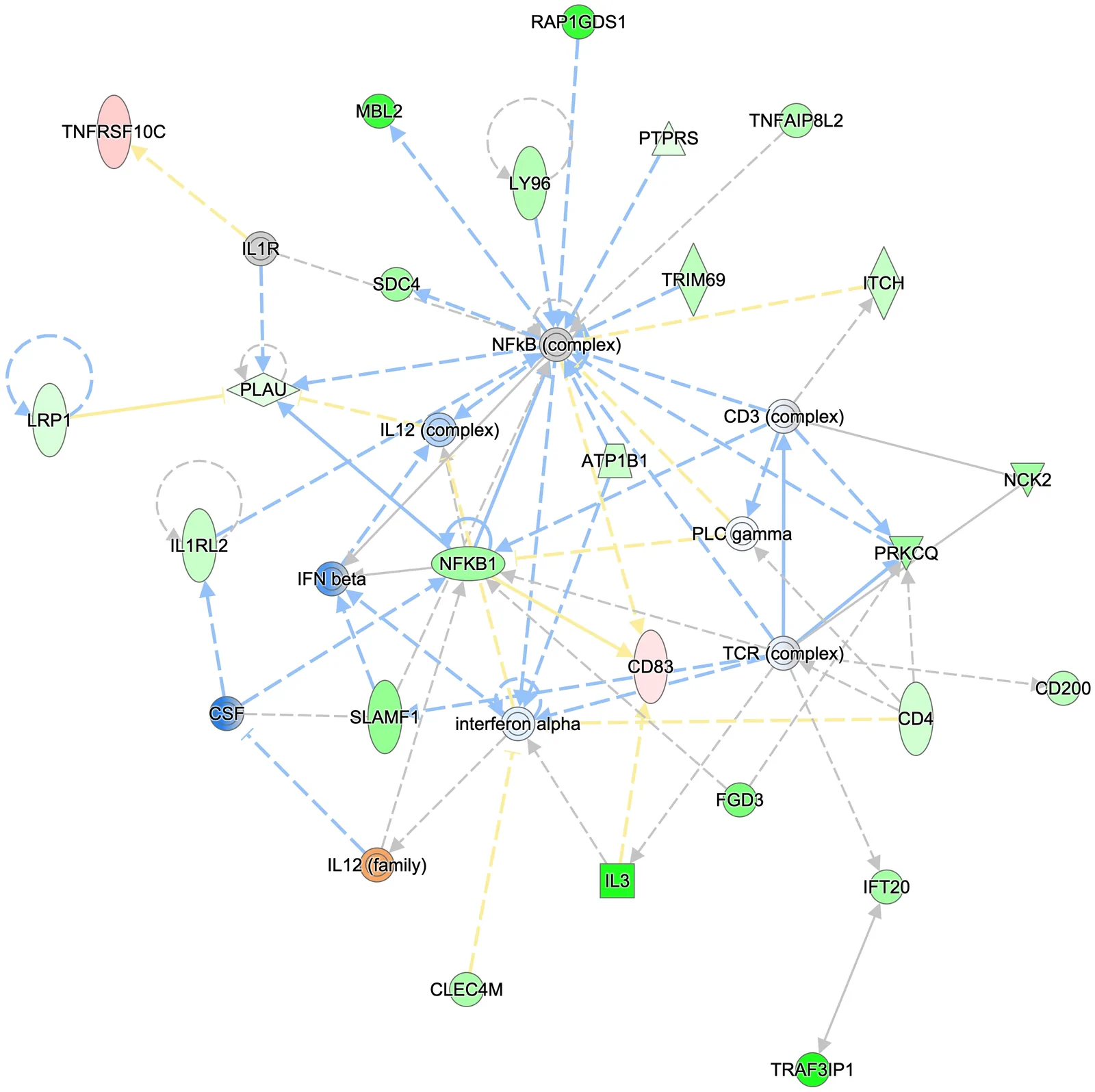
Network of cell-to-cell signaling and interaction, hematological system development and function, inflammatory disease. Node color and intensity reflect the observed degree of gene expression dysregulation, with red (upregulation) and green (downregulation). Predicted activation states of genes within the network are shown in orange (activated), blue (inhibited), and gray (not predicted), with relationships represented by solid lines (direct) or dashed lines (indirect). Lines in orange, blue, yellow, and gray indicate relationships between nodes that lead to activation, inhibition, inconsistent findings, and an unexpected effect, respectively.

## 4 Discussion

This discovery-based study focused on identifying candidate proteins and biological pathways that may elucidate novel candidate protein biomarkers as well as novel underlying protein pathways associated with chronic PTSD symptoms. Our findings are consistent with previous findings of a significant role for inflammatory pathways associated with PTSD and related psychological conditions, and of our current understanding of these significantly differentially expressed proteins/protein pathways in PTSD. However, our findings are discovery-focused and considered hypothesis-generating rather than confirmatory or diagnostic. Although individual protein changes were subtle, systems-level analysis revealed downregulation of ECM and vascular remodeling and diminished immune cell activation, suggesting potential biological mechanisms that may be related to vascular inflammation and endothelial dysfunction that can lead to broader vascular and brain disease in the long term.

Although differences in individual protein expression did not reach statistical significance in adjusted p-values, the observed patterns align with and can be contextualized within the existing literature. Downregulation of Leiomodin-2 (LMOD2), a regulator of actin filament assembly (log2FC= -0.53, p=4.64E-04), and ATP synthase δ-subunit (ATP5F1D), a subunit of mitochondrial ATP synthesis (log2FC= -0.79, p=9.34E-04), suggests involvement of cytoskeletal remodeling and mitochondrial energy metabolism in PTSD, though their direct functional relevance to PTSD warrants further investigation. CASKIN1 and Neuroligin-1 (NLGN1) were downregulated in participants with PTSD (log2FC= -1.17 and -0.56, p=1.38E-03 and 1.40E-03, respectively), suggesting their biological relevance in PTSD and chronic stress response. CASKIN1 is a neuronal scaffolding protein exclusively expressed in brain tissue, which helps organize the machinery at the junction of synapses (21). CASKIN1 has been previously identified as a potential marker of synaptic damage in stroke pathology (22) and stress response (23). NGLN1 is a postsynaptic adhesion molecule that binds to β-neurexins (24). It has been linked to PTSD through a genome-wide gene-based analysis (25), and plays a key role in how the brain forms and strengthens fear memories (26). Another protein, T-cell intracellular antigen 1 (TIA1), was downregulated in the PTSD-Present group (log2FC=-0.71, p=2.07E-02). TIA1 is an RNA-binding protein that regulates gene expression under cellular stress. Its relevance to PTSD is supported by evidence linking TIA1 to fear memory and fear extinction in mice (27, 28) and anxiety symptoms in humans (29), suggesting that our findings support the role of these peripheral proteins in possible central activities.

In the pathway analysis, we observed downregulation of ECM organization, VEGF signaling, and wound-healing pathways, suggesting reduced vascular remodeling and heightened blood-brain barrier (BBB) vulnerability may be associated with chronic PTSD. In addition to providing a structural scaffold for the neurovascular unit, ECM is a dynamic regulator of synaptic plasticity, as perineuronal nets stabilize synaptic connections (30). Together, downregulation of the pathways may be linked to the cognitive and emotional deficits central to PTSD, especially in chronic PTSD. These differences in protein abundance in PTSD also suggest that these structural components are frequently implicated in the maladaptive response to chronic stress (31), and also may be potentially hindering the neural reorganization required for successful fear extinction and recovery, and possibly contributing to PTSD vulnerability, or chronicity, or both. Furthermore, the reduced signaling of VEGF and Platelet-Derived Growth Factor (PDGF) may be important to consider, as both are essential for angiogenesis and tissue regeneration, and align with previous findings from a multi-omics analysis of combat-exposed service members with PTSD (32), which reported impaired angiogenesis to be associated with PTSD. Relatedly, the inhibition of wound-healing processes observed in our cohort aligns with previous evidence identifying these pathways as primary biological targets of PTSD-related differences in service members (32). Ultimately, following validation of these findings, these convergent pathways may point toward a breakdown of the neurovascular unit and potentially suggest a fundamental impairment of structural plasticity in chronic PTSD.

The findings from the network analysis also showed that NFκB, a critical regulator of immune response, cellular response, and stress, is potentially related to PTSD symptom burden. Specifically, NFκB1, a key DNA-binding component of the NFκB complex, was downregulated in the PTSD-Present group. Further, concurrent suppression of immune and inflammatory signaling pathways was observed, including macrophage activation, CGAS-STING, High Mobility Group Box 1 (HMGB1), and interleukin-17 (IL-17) signaling pathways. These findings suggest that a coordinated downregulation of immune-related transcription factors and pathways may indicate reduced immune activation and a suppressed inflammatory response among individuals with PTSD (33–35). This reduced inflammatory and immune response possibly influences broader health outcomes among individuals with PTSD, which should be examined in future studies after validation of these differentially expressed proteins and protein pathways. A study of 660,000 Iraq and Afghanistan veterans reported that veterans with PTSD are twice as likely to suffer from autoimmune disorders as veterans with no psychiatric diagnosis (36), suggesting that this may be a consequence of suppressed immune systems. Furthermore, decreased immune response in PTSD contributes to early cognitive decline and cardiovascular disease (37). As the current study examined peripheral plasma and did not specifically assess immune cell counts and composition, it is unclear whether these proteins exhibit central activity. Thus, further studies are warranted to investigate the mechanisms underlying differential immune response in PTSD.

Taken together, these findings suggest potential novel proteins and pathways associated with PTSD, including dysregulation in vascular, immune, and synaptic systems, providing candidate pathways for future mechanistic and biomarker studies. This may also be associated with individual PTSD symptoms and PTSD-related negative long-term health outcomes, including autoimmune (38), cardiovascular (39), metabolic (6), and neurodegenerative (40) disorders. Further studies to validate these exploratory findings are warranted, including traditional protein quantification, replication in independent cohorts, and multi-omics approaches.

### 4.1 Strengths and limitations

Beyond the discovery candidate-protein approach, the use of an extensive, unbiased panel of proteins enabled exploration of novel proteins and protein pathways that may serve as biomarkers and generate hypotheses regarding the physiological underpinnings of PTSD symptom burden in SMVs. While these discovery findings require replication, quantification, and validation of novel candidate PTSD biomarkers, these findings could potentially lead to the development of novel targeted treatments for individuals with PTSD, refractory to current available treatments. Future studies should complete similar analyses in larger samples and include additional analyses for different symptom clusters, as the pathophysiological processes underlying unique symptoms of PTSD (e.g., hyperarousal) may differ from those of mood-related symptoms.

The present study is not without limitations. Importantly, while this represents one of the largest military PTSD biomarker studies to date, the sample size (n=208) was relatively small in relation to the number of proteins assayed. Based on the *post-hoc* power analysis, the study was adequately powered to detect large effects, but likely underpowered for the smaller effect size. Relatedly, the differential analysis for individual protein findings did not survive FDR correction for multiple comparisons. However, this study also aimed to discover related pathways that might generate systems-level insights and was not focused on individual protein expression differences; this enabled us to detect subtle but meaningful changes across pathways. Reporting pathways that survived FDR correction and Z-score criteria for significance in the pathway analyses reduced false discovery. Moreover, methodologically, Olink proximity extension assay relies on antibody-pair specificity rather than direct peptide detection, as in mass spectrometry. Therefore, the findings from this study may not be directly generalizable across studies using different platforms due to platform dependence (41).

Other limitations include our use of a PTSD screening tool (PCL-C) to determine the PTSD-present group rather than a clinical interview, such as the Clinician-Administered PTSD Scale for DSM-5 (CAPS-5), which may result in less sensitive diagnostic measurement. Further, the present study examined total symptom burden scores as the outcome variable rather than symptom clusters. PTSD is a heterogeneous disorder, and the underlying neurobiology of different symptom clusters may vary. Moreover, we did not account for other mental and physical comorbidities or treatment, including prescription medications, which can also influence protein expression levels. Particularly, mTBI and PTSD frequently co-occur and share overlapping biological mechanisms (42, 43); we did not statistically adjust for them to prevent the risk of obscuring the genuine shared signal, which limits our ability to isolate TBI-related effects. Additionally, the plasma proteome reflects systemic biology and fluid inflammation, which limits the ability to link findings to brain biology or neuro-specific changes, even though some proteins play roles related to neurotransmitters and synapses. Lastly, the present sample consists of SMVs who self-reported as White at one clinical site and therefore may not generalize to other groups or the general population.

## Data Availability

The proteomics dataset generated in this study has been deposited in the PRIDE repository through the ProteomeXchange Consortium under accession number PAD000055. The dataset is available at https://www.ebi.ac.uk/pride/archive/projects/PAD000055. De-identified summary/aggregate data will be provided on request. However, individual data elements are not available due to DoD legal requirements and current IRB-approved language in the subject consent forms.
